# Resource Quantity Affects Benthic Microbial Community Structure and Growth Efficiency in a Temperate Intertidal Mudflat

**DOI:** 10.1371/journal.pone.0038582

**Published:** 2012-06-18

**Authors:** Daniel J. Mayor, Barry Thornton, Alain F. Zuur

**Affiliations:** 1 Institute of Biological and Environmental Sciences, Oceanlab, University of Aberdeen, Aberdeen, United Kingdom; 2 The James Hutton Institute, Craigiebuckler, Aberdeen, United Kingdom; 3 Highland Statistics Ltd., Newburgh, Aberdeenshire, United Kingdom; Argonne National Laboratory, United States of America

## Abstract

Estuaries cover <1% of marine habitats, but the carbon dioxide (CO_2_) effluxes from these net heterotrophic systems contribute significantly to the global carbon cycle. Anthropogenic eutrophication of estuarine waterways increases the supply of labile substrates to the underlying sediments. How such changes affect the form and functioning of the resident microbial communities remains unclear. We employed a carbon-13 pulse-chase experiment to investigate how a temperate estuarine benthic microbial community at 6.5°C responded to additions of marine diatom-derived organic carbon equivalent to 4.16, 41.60 and 416.00 mmol C m^−2^. The quantities of carbon mineralized and incorporated into bacterial biomass both increased significantly, albeit differentially, with resource supply. This resulted in bacterial growth efficiency increasing from 0.40±0.02 to 0.55±0.04 as substrates became more available. The proportions of diatom-derived carbon incorporated into individual microbial membrane fatty acids also varied with resource supply. Future increases in labile organic substrate supply have the potential to increase both the proportion of organic carbon being retained within the benthic compartment of estuaries and also the absolute quantity of CO_2_ outgassing from these environments.

## Introduction

Estuaries are net heterotrophic systems [1], [2] and represent a significant source of CO_2_ to the atmosphere: Regional-scale estimates suggest that European estuaries represent 5–10% of Western Europe’s anthropogenic CO_2_ emissions [3]. The exact contribution of estuaries to the global atmospheric CO_2_ emissions remains contentious, but estimates range between 0.25–0.45 Pg C y^−1^ [2], [4]. Bacteria play a pivotal role in the mineralization of organic matter along estuaries, with CO_2_ emissions from these ecosystems originating principally from bacterial respiration [2], [5]. They are also fundamental for mediating changes in the nitrogen cycle [6], and thereby influence the availability of key nutrients for primary producers. It follows that understanding the processes controlling microbial mineralization in estuarine waterways is a prerequisite for predicting the future role of this habitat in global elemental cycles and thus climate regulation. Estuarine sediments can efficiently bury organic matter, sequestering both carbon and nitrogen from the atmosphere [1], [7], [8]. A deeper appreciation of estuarine biogeochemistry is therefore also required if we are to forecast how the production and storage of organic matter in coastal ecosystems will be affected by further anthropogenic change.

Agricultural practices and human wastewaters both contribute significantly to the eutrophication of rivers and coastal systems [9], [10], resulting in a greater supply of labile organic substrates to the seabed. Work conducted on bacterioplankton communities has demonstrated that the rates and efficiencies with which they grow are both positively related to substrate availability [11]–[13]. A positive relationship between benthic estuarine mineralization rates and carbon input also exists when multiple locations are considered [1]. Far less is known about how increased organic matter supply affects benthic microbial community structure and function within a single estuary. Indeed, estimates of estuarine benthic bacterial growth efficiency (BGE) and the factors controlling it are scarce [14]. We used a carbon-13 (^13^C) tracer study to explore the hypothesis that substrate quantity affects the composition and short-term metabolic response of the benthic bacterial community in a temperate estuary. Increasing quantities of ^13^C-labelled diatoms were added to hand-collected sediment cores, allowing us to quantify the amounts of diatom-derived carbon that were mineralized and incorporated into benthic bacterial biomass during the experiment. Our results demonstrate that carbon mineralization, bacterial biomass production and BGE are all coupled to the supply of labile substrates, illustrating that resource quantity plays a key role in controlling the short-term fate of organic matter in temperate estuarine sediments.

## Materials and Methods

### Study Site and Sediment Collection

The experiment was conducted on natural whole-sediment communities retrieved from the tidal mudflats in the lower reach of the Ythan Estuary, Aberdeenshire, Scotland, UK (57°20.085′N, 02°0.206′W). All necessary permissions for work on the Ythan and Forvie National Nature Reserve were obtained from Scottish Natural Heritage. The sediments at the experimental location have a mean particle diameter of 50 µm [15] and contained 1.5% organic carbon by dry weight in the upper 1 cm. A total of 12 Perspex cores (10 cm ID×600 mm length) were inserted 12 cm into the sediments by hand at low tide and the resulting material retrieved. All sediment cores were transferred to a temperature controlled laboratory, set at the in situ temperature of 6.5°C, within 30 minutes of collection. Each core subsequently received 3.8 L of UV-sterilized, 10 µm filtered seawater previously collected from the estuary at high tide (∼33 psu).

### Experimental Setup

The experiment consisted of four treatments, with three replicates of each: Control (no substrate addition), low-, medium-, and high-quantity of organic material. Sediments in the latter three treatments received the equivalent of 4.16, 41.60 and 416.00 mmol organic carbon m^−2^ respectively in the form of a ^13^C-labelled marine diatom, *Chaetoceros radicans* (49.4±0.3 atom % ^13^C). These quantities of carbon were chosen to fall below and above 11.42 mmol organic carbon m^−2^, the mean daily amount of phytoplankton estimated to be deposited on Ythan sediments [16]; the three levels of organic enrichment are representative of the mean daily quantities of carbon received by oligotrophic, mesotrophic and hypertrophic estuaries respectively [17]. The use of ^13^C-labelled *C. radicans* enabled us to trace the fate of the constituent carbon into dissolved inorganic carbon (DI^13^C) and bacterial biomass in a quantitative manner. Specific details of algal culture techniques and biochemistry of the *C. radicans* are presented elsewhere [18]. Immediately prior to experimentation, the algal substrates were suspended in 10 ml of seawater and gently pipetted directly onto the sediment surface to ensure homogenous distribution. All cores were subsequently sealed with lids to prevent gas exchange and incubated in darkness. Water samples from each core, collected via lid ports immediately after the introduction of substrates (t = 0) and every 4 hours thereafter, were analysed for concentrations of dissolved oxygen, DIC and DI^13^C, ammonium-nitrogen (NH_4_-N) and total oxidised nitrogen (TOx-N; NO_2_+NO_3_). Core lids were gently depressed into the cores as the water samples were drawn to avoid the production of a head space. Stirrer-bars that passed through the lids via an o-ring seal were rotated immediately prior to each sampling interval without disturbing the sediment surface to ensure that the water was well homogenized. Sediments were extruded at the end of the experiment and the upper 1 cm was retained and stored at −80°C for subsequent quantification of diatom carbon uptake into bacterial biomass.

### Sample Processing

Samples for determination of oxygen concentrations were transferred into 10 ml Winkler bottles, fixed and subsequently analysed using an automated Winkler titration system (785 DMP Titrino, Metrohm U.K.). Concentrations of NH_4_-N and TOx-N were determined using an automated segmented flow analyser (Bran & Leubbe QuAAtro SFA, SEAL Analytical Ltd., U.K.). Aliquots of water for the analysis of DIC and DI^13^C were sterile filtered (0.2 µm) into Exetainers (Labco, U.K.), poisoned with 0.2% (vol) mercuric chloride and stored at 4°C until analysis. DIC samples were quantitatively converted to carbon dioxide before the concentrations and carbon isotope ratios were determined using a Gas-bench II connected to a Delta^Plus^ Advantage isotope ratio mass spectrometer (IRMS; both Thermo Finnigan, Germany) [19]. The mean amplitude of five replicate sample peaks was used to calculate DIC concentration from a calibration curve derived from an appropriate range of sodium carbonate standard solutions.

Purified phospholipid fatty acids (PLFAs) extracted from freeze-dried sediment samples [20], [21] were derivitized to yield fatty acid methyl esters (FAMEs). The concentrations and carbon isotope ratios of individual FAMEs were measured using a GC Trace Ultra with combustion column attached via a GC Combustion III to a Delta V Advantage IRMS (all Thermo Finnigan, Germany). Individual PLFAs were quantified by combining the area of their mass peaks, m/z = 44, 45 & 46, after background subtraction, and comparison with a known internal standard (19:0) added to each sample [22]. Bacterial carbon uptake was calculated from label incorporation into the bacterial biomarker PLFAs i15:0, ai15:0 and i16:0 [23], assuming these represent 10% of total bacterial PLFAs and 0.056 gC PLFA/gC biomass [24]. All calculations relating to the uptake of ^13^C were made using well-established equations [23]. Data are expressed as the total uptake and mineralization of added diatom-derived carbon (^12^C +^13^C). Bacterial growth efficiency (BGE) was estimated as: *I_B_/(I_B_+R_B_)*, where *I_B_* and *R_B_* are the quantities of diatom-derived carbon incorporated into bacterial biomass and respired over the duration of the experiment respectively. The resulting estimates are considered to be minimum estimates as a proportion of the quantified respiration may have been attributable to metazoan organisms (see Discussion).

### Statistical Analyses

All statistical analyses were conducted in the ‘R’ programming environment [25] using the ‘nlme’ and ‘MASS’ packages [26], [27]. Repeated seawater sampling from each core necessitated that all of the resulting benthic flux data were analysed using linear mixed-effects (LME) models that included core identity as a random effect [28]. Variance covariate terms were also incorporated in the random structure of the models in instances of unequal variances. The fixed structures of the statistical models initially incorporated time and treatment and an interaction between these terms. Backwards model selection, based on the likelihood ratio test using maximum likelihood estimation, was employed to determine the fixed structures of the optimal models (OMs) [28], [29]. Restricted maximum likelihood estimation was used to generate model parameter estimates. All OMs were validated to check that the underlying assumptions were met: Normality of residuals was examined by plotting theoretical quantiles versus standardized residuals (Q-Q plots); homogeneity of variance was assessed by plotting residual versus fitted values; independence was verified by plotting residuals versus each covariate [28]. Estimated values±standard errors (se) are presented.

Bacterial carbon uptake and BGE data were box-cox transformed to attain homogeneity of variance prior to analysis using one-way analysis of variance (ANOVA). Post-hoc multiple comparisons were achieved using Tukey’s honest significant difference tests. Treatment effects on the proportional uptake of diatom-derived carbon into individual PLFAs, a relative indication of the structure of the active microbial community [30], were examined using correlation-based principle components analysis [31]. The PLFAs 14:0, 16:1(n-7), 16:1(n-5), 16:0 and all C18s were excluded from this analysis owing to their prevalence in the diatoms [18].

## Results

### Benthic Fluxes

**Figure 1:**
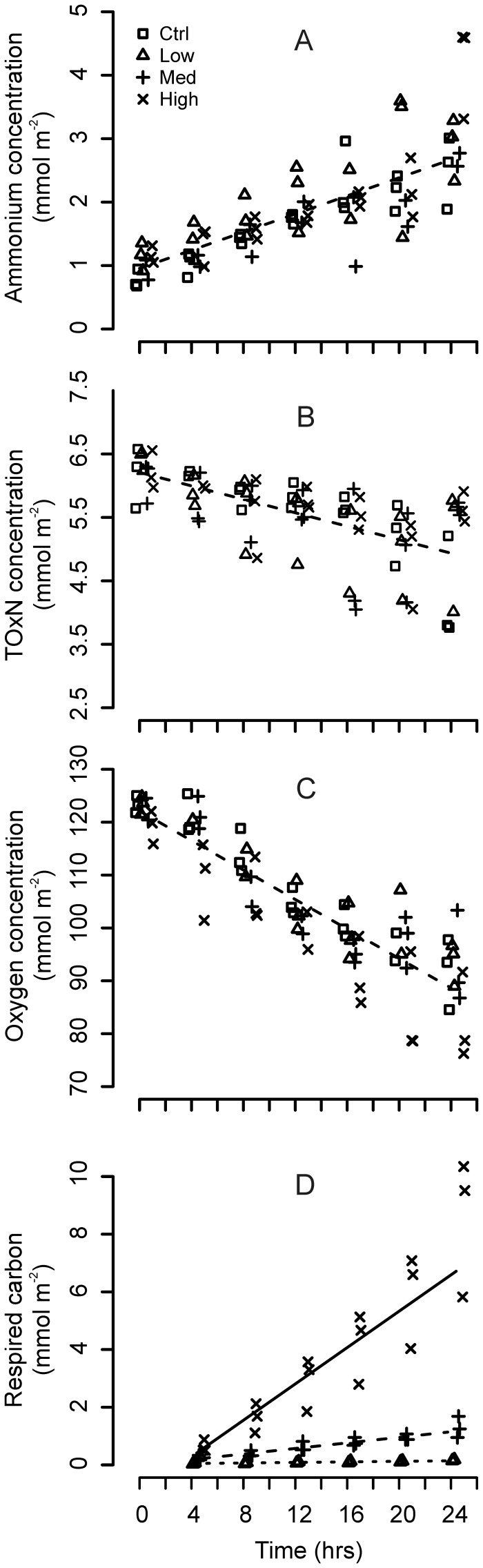
Resource-quantity effects on benthic fluxes. Temporal trends in the concentrations of NH_4_-N (A), TOx-N (B), oxygen (C) and respired diatom-derived carbon (D). Data from the control, low-, medium- and high-treatments are represented by squares, triangles, vertical- and diagonal crosses respectively.

Concentrations of NH_4_-N and TOx-N were inversely related (Figs. 1A and 1B): NH_4_-N increased at a rate of 1.71 mmol ±0.11 m^−2^ d^−1^ (L.Ratio = 105.44, df_1_, p<0.001; Table S1) and TOx-N decreased at a rate of 1.27±0.15 mmol m^−2^ d^−1^ (L.Ratio = 50.90, df_1_, p<0.001; Table S2). These rates were not affected by the quantities of added diatoms (Time×Treatment interactions; L.Ratios<4.5, df_3_, p>0.21 in both cases). Oxygen concentrations declined significantly over time (Fig. 1C; L.Ratio = 146.91, df_1_, p<0.001; Table S3) but drawdown rates were not affected by the quantity of added diatoms (Time×Treatment interaction; L.Ratio = 5.09, df_3_, p = 0.165); oxygen was consumed at a rate of 33.45±1.43 mmol m^−2^ d^−1^. In contrast, the rate at which diatom-derived carbon was mineralized increased significantly with increasing quantities of added material (Fig. 1D; Time×Treatment interaction; L.Ratio = 146.73, df_2_, p<0.001; Table S4); mineralization rates in the low, medium and high treatments are presented in Table 1.

**Table 1:** The effect of resource-quantity on estuarine benthic carbon budgets.

	Resource quantity		
	Low	Medium	High
**Mineralization**	0.143±0.05 (3.4)	1.149±0.06 (2.8)	6.589±0.50 (1.6)
**Bacterial uptake**	0.096±0.01 (2.3)	1.314±0.12 (3.2)	8.201±1.51 (2.0)
**BGE**	0.40±0.02	0.53±0.02	0.55±0.04

Mineralization and uptake units are mmol C m^−2^ d^−1^±SEM. Estimated bacterial growth efficiencies (BGE) are expressed as proportions±SEM. Values in parentheses represent the percentage of total carbon added.

### Microbial Carbon Processing

**Figure 2:**
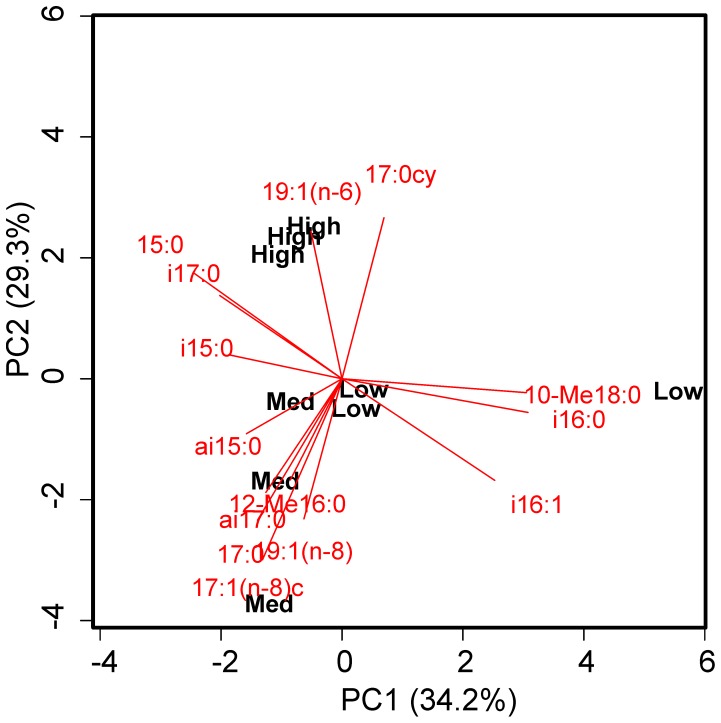
Influence of resource availability on carbon uptake into individual phospholipid fatty acids. Principle components analysis distance biplot visualising differences in the proportional uptake of ^13^C into the phospholipid fatty acids between the different treatments.

The quantities of diatom-derived carbon incorporated into bacterial biomass at the end of the experiment increased significantly with the quantity of material added (Table 1; F = 436.45, df_2,6_, p<0.001). Treatment effects on BGE were also apparent (Table 1; F = 10.25, df_2,6_, p = 0.012). The proportional uptake of diatom-derived carbon into individual fatty acids differed by treatment (Fig. 2). The microbial community in the low treatment discriminated on the first principal component (PC1), with positive loadings of 10-Me18:0, i16:0 and i16:1 and negative loadings of 15:0, i15:0 and i17:0. Communities in the medium and high treatments discriminated on the second principal component (PC2); the former was characterised by increased carbon uptake into 17:1(n-8)c, 17:0, 19:1(n-8), ai17:0 and 12-Me16:0 and decreased uptake into 17:0cy and 19:1(n-6). The inverse pattern was observed in the high treatment.

## Discussion

Our measured fluxes of NH_4_-N, TOx-N and oxygen (Fig. 1A, B and C) are in good agreement with earlier observations from other temperate, intertidal mudflat sediments [32]–[36]. The null effect of resource supply on these fluxes (p>0.16 in all cases; Fig. 1) is consistent with previous benthic enrichment studies in which relatively small quantities of organic carbon have been added [23], [37], [38]. These data do not imply that oxygen and nitrogen cycle independently of the amount of organic carbon available; it is well known that the deposition of excessive quantities of organic material on the seabed impact upon benthic fluxes of oxygen and nitrogen [39]. Rather, they demonstrate that our substrate additions were sufficiently low to avoid driving a major change to the natural functioning of the benthos. It follows that the majority of the oxygen consumed during our incubations was used to catabolise substrates other than those introduced experimentally and/or for the reoxidation of reduced inorganic metabolites produced during anaerobic respiration [40]. Similarly, the observed nitrogen fluxes relate to the cycling of autochthonous substrates, and not necessarily to the activities of the microbial communities responding to the diatom additions.

Resource-availability had a significant, stepwise effect on the quantities of diatom-derived carbon that were mineralized (Fig.1D) and incorporated into bacterial biomass (Table 1). These data support the understanding that the catabolism of organic matter in marine sediment ecosystems, from tropical estuaries and Arctic shelf sediments to the deep-sea, is directly related to the quantity of labile respiratory substrates [38], [41]–[44]. Previous studies using two levels of ^13^C-enriched diatoms to investigate benthic carbon cycling also reported a similar, stepwise effect of substrate availability on mineralization rates in coastal and deep-sea sediment ecosystems [45], [46]. Our study illustrates the sensitivity of ^13^C tracer experiments relative to measuring bulk ecosystem parameters such as oxygen consumption. It also highlights the need for caution when attempting comparisons between different ^13^C tracer studies; the outcome of such experiments clearly depends upon the quantity and quality of the available substrates [38], [45], [46].

Estimated BGEs increased significantly with the supply of labile substrates (Table 1), despite the temperature being well below the seasonal maximum of ∼20°C. Our estimates of BGE fall within previously observed values in estuarine and deep-sea sediments [14], [37], [38] and agree closely with the value of 0.5 observed for a natural marine bacterioplankton community growing on diatom aggregates [47]; they also correspond with an apparent plateau in BGE of ∼0.5 observed across a range of eutrophic pelagic systems [48]. Benthic metazoans, particularly nematodes, contribute significantly to carbon mineralization in estuarine sediments [1], [49]. We did not quantify metazoan contributions to mineralization processes, therefore our values of BGE may be considered to be minimal estimates. However, a growing number of studies report negligible mineralization and uptake of diatom-derived carbon by nematodes during short-term tracer incubation studies [37], [50]–[52]. This suggests that they are not directly involved in the catabolism of detrital material, at least within the time-scale of the present study. Close agreement between the BGEs presented herein and previous estimates suggests that the metazoan contribution to carbon mineralization in our experiments was low. This interpretation is further supported by other work which indicates that estuarine detrital carbon cycling is predominated by bacteria whereas the constituent metazoans feed selectively on living, autocthonous microphytobenthos [53]. Our observations indicate that the growth of the bacterial community in our experimental sediments was directly regulated by resource availability, as previously reported for bacterioplankton communities [11]–[13]. The positive relationship between BGE and resource supply reflects a progressive uncoupling between bacterial biomass production and respiration. This finding is consistent with theory and previous observations; a greater proportion of assimilated resources must be allocated to meet basal demands for biomass maintenance (as opposed to growth) when resources are scarce [12], [13], [54].

The relative uptake of tracer carbon into the different PLFAs examined in our study changed between the low, medium and high substrate additions (Fig. 2), likely reflecting a range of complex and interacting processes. Previous work using terminal restriction fragment length polymorphism analysis found no effect of resource supply on the prokaryotic community composition of tidal creek sediments [55]. Similarly, there were no appreciable changes in the proportional uptake of ^13^C into different PLFAs in a deep-sea sediment community when exposed to two different quantities of ^13^C-enriched diatoms [46]. Differences in the relative distribution of ^13^C-labelling between the treatments in our experiment may therefore reflect a resource-dependent change in the balance between catabolism and anabolism of individual PLFAs within the active component of the bacterial community; the relative abundance of certain PLFAs are known to be affected by external stressors [56], [57] and can change in response to the substrates used for biosynthesis [58]. However, the most dominant factors discriminating between the low-, medium- and high-treatments, 10Me-18:0, 17:1(n-8) and 17:0cy respectively, are typical of sulfate-reducing bacteria [56], [59]. This group of organisms is responsible for approximately 50% of all carbon degradation in shallow water sediments [60]. They grow under anaerobic conditions but are capable of aerobic carbon mineralization [61], [62]. We therefore suggest that the observed changes in ^13^C uptake into individual PLFAs predominantly reflects a progressive shift towards a sulfate-reducing microbial community as substrate supply increased [56]. However, the present data do not allow us to conclusively differentiate between the suggested explanations. The isotope-based PLFA technique is a powerful and sensitive method for discerning carbon uptake in natural microbial communities. The effectiveness of this approach is, however, tempered by an inability to differentiate between a true shift in the microbial community structure and metabolic changes within the same community owing to the poor specificity of individual biomarker PLFAs. Irrespective of the underlying mechanism, substrate-induced shifts in the synthesis of individual compounds has implications for the energetic and nutritional value of estuarine sediments to the communities of deposit feeding animals that inhabit them.

In conclusion, resource quantity had a profound effect on the rates of carbon mineralization and uptake into specific PLFAs in a temperate estuarine sediment microbial community. Processes that increase the supply of labile resources to this environment will result in a greater proportion of the organic carbon being retained in the benthic food web due to increased bacterial growth efficiency. Nevertheless, the absolute quantities of CO_2_ resulting from microbial mineralization will increase with the input of labile organic matter, at least over the range investigated herein. More work is needed to refine our understanding of the longer-term impacts of resource availability on microbial community structure and functioning and the implications for stocks of previously sequestered carbon in estuarine sediments.

## Supporting Information

Table S1
**Model output from the NH_4_-N concentration data analysis.** The optimal model (OM) was a LME model that incorporated core identity as a random effect (L. ratio = 14.230, df_1_, p_corr_<0.001) and allowed the residual spread to increase exponentially over time (L. ratio = 44.507, df_1_, p<0.001): 
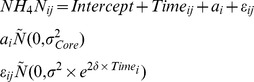
 where *a_i_* is a random intercept and the index *i* refers to the core identity (*i* = 1,…, 12), and *j* to the observations within each core (*j* = 1,…,7). Random effect (a), variance function (b), correlation coefficients of observations made within each variance grouping (intra-class correlation) and fixed effects (d). ^*^Note the intercept (baseline) is the control treatment.(DOC)

Table S2
**Model output from the TOx-N concentration data analysis.** The optimal model (OM) was a LME model that incorporated core identity as a random effect (L. ratio = 5.390, df_1_, p_corr_ = 0.010) and allowed the residual spread to increase exponentially over time (L. ratio = 15.366, df_1_, p<0.001): 
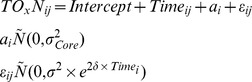
 where *a_i_* is a random intercept and the index *i* refers to the core identity (*i* = 1,…, 12), and *j* to the observations within each core (*j* = 1,…,7). Random effect (a), variance function (b), correlation coefficients of observations made within each variance grouping (intra-class correlation) and fixed effects (d). ^*^Note the intercept (baseline) is the control treatment.(DOC)

Table S3
**Model output from the oxygen concentration data analysis.** The optimal model (OM) was a LME model that incorporated core identity as a random effect (L. ratio = 19.467, df_1_, p<0.001): 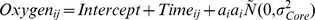 where *a_i_* is a random intercept and the index *i* refers to the core identity (*i* = 1,…, 12), and *j* to the observations within each core (*j* = 1,…,7). Random effect (a), correlation coefficients of observations made within each core [intra-class correlation] (b) and fixed effects (c). ^*^Note the intercept (baseline) is the control treatment.(DOC)

Table S4
**Model output from the DIC concentration data analysis.** The optimal model (OM) was a LME model that incorporated core identity as a random effect (L. ratio = 48.237, df_1_, p_corr_<0.001) and allowed the residual spread to increase exponentially over time and to vary by treatment (L. ratio = 179.335, df_3_, p<0.001): 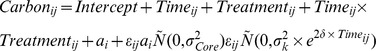 where *a_i_* is a random intercept and the index *i* refers to the core identity (*i* = 1,…, 9), *j* to the observations within each core (*j* = 1,…,6) and *k* to the treatment (*k* = 1,…, 3). Random effect (a), variance function (b), correlation coefficients of observations made within each variance grouping (intra-class correlation) and fixed effects (d). ^*^Note the intercept (baseline) is the low diatom-addition treatment.(DOC)
