# Supplementary material for: Resource Quantity Affects Benthic Microbial Community Structure and Growth Efficiency in a Temperate Intertidal Mudflat
Source: PLoS One. 2012 Jun 18;7(6):e38582. doi: 10.1371/journal.pone.0038582 (PMC3377660; doi:10.1371/journal.pone.0038582)
Supplement: Table S1 — Model output from the NH4-N concentration data analysis. The optimal model (OM) was a LME model that incorporated core identity as a random effect (L. ratio = 14.230, df1, pcorr<0.001) and allowed the residual spread to increase exponentially over time (L. ratio = 44.507, df1, p<0.001): where ai is a random intercept and the index i refers to the core identity (i = 1,…, 12), and j to the observations within each core (j = 1,…,7). Random effect (a), variance function (b), correlation coefficients of observations made within each variance grouping (intra-class correlation) and fixed effects (d). *Note the intercept (baseline) is the control treatment. (DOC) [file pone.0038582.s001.doc]

**Table S1. Model output from the NH_4_-N concentration data analysis.** The optimal model (OM) was a LME model that incorporated core identity as a random effect (L. ratio = 14.230, df_1_, p_corr_ < 0.001) and allowed the residual spread to increase exponentially over time (L. ratio = 44.507, df_1_, p < 0.001):

$$\begin{matrix} NH_{4}N_{ij}=Intercept+Time_{ij}+a_{i}+\varepsilon_{ij} \\ a_{i}\sim N(0,\sigma_{Core}^{2}) \\ \varepsilon_{ij}\sim N(0,\sigma^{2}\times e^{2\delta\times Time_{i}}) \end{matrix}$$

where *a_i_* is a random intercept and the index *i* refers to the core identity (*i* = 1,..., 12), and *j* to the observations within each core (*j* = 1,...,7). Random effect (a), variance function (b), correlation coefficients of observations made within each variance grouping (intra-class correlation) and fixed effects (d). ^*^Note the intercept (baseline) is the control treatment.

| (a) | **Model term** | **σ** | |  |  |  |
| --- | --- | --- | --- | --- | --- | --- |
|  | Core ID | 0.203 | |  |  |  |
| (b) | **Variance term** | **Variance estimates** | |  |  |  |
|  | δ | 0.080 | |  |  |  |
|  | Time | $0.109^{2}\times e^{2\times0.080\times Time_{i}}$ | |  |  |  |
|  |  | **Intra-class correlation** | | |  |  |
| (c) | Time (hrs) |  |  |  |  |  |
|  | 4 | 0.999 |  |  |  |  |
|  | 8 | 0.999 |  |  |  |  |
|  | 12 | 0.999 |  |  |  |  |
|  | 16 | 0.998 |  |  |  |  |
|  | 20 | 0.995 |  |  |  |  |
|  | 24 | 0.991 |  |  |  |  |
| (d) | **Model term** | **Value ± SE** | | **df** | **t** | **p** |
|  | Intercept^*^ | 0.957 ± 0.068 | | 64 | 13.975 | <0.001 |
|  | Time | 0.071 ± 0.004 | | 64 | 16.100 | <0.001 |
