# Supplementary material for: Resource Quantity Affects Benthic Microbial Community Structure and Growth Efficiency in a Temperate Intertidal Mudflat
Source: PLoS One. 2012 Jun 18;7(6):e38582. doi: 10.1371/journal.pone.0038582 (PMC3377660; doi:10.1371/journal.pone.0038582)
Supplement: Table S3 — Model output from the oxygen concentration data analysis. The optimal model (OM) was a LME model that incorporated core identity as a random effect (L. ratio = 19.467, df1, p<0.001): where ai is a random intercept and the index i refers to the core identity (i = 1,…, 12), and j to the observations within each core (j = 1,…,7). Random effect (a), correlation coefficients of observations made within each core [intra-class correlation] (b) and fixed effects (c). *Note the intercept (baseline) is the control treatment. (DOC) [file pone.0038582.s003.doc]

**Table S3. Model output from the oxygen concentration data analysis.** The optimal model (OM) was a LME model that incorporated core identity as a random effect (L. ratio = 19.467, df_1_, p < 0.001):

$$\begin{matrix} Oxygen_{ij}=Intercept+Time_{ij}+a_{i} \\ a_{i}\sim N(0,\sigma_{Core}^{2}) \end{matrix}$$

where *a_i_* is a random intercept and the index *i* refers to the core identity (*i* = 1,..., 12), and *j* to the observations within each core (*j* = 1,...,7). Random effect (a), correlation coefficients of observations made within each core [intra-class correlation] (b) and fixed effects (c). ^*^Note the intercept (baseline) is the control treatment.

| (a) | **Model term** | **σ** | |  |  |  |
| --- | --- | --- | --- | --- | --- | --- |
|  | Core ID | 4.578 | |  |  |  |
| (b) |  | **Intra-class correlation** | | |  |  |
|  | Core ID | 0.552 |  |  |  |  |
| (d) | **Model term** | **Value ± SE** | | **df** | **t** | **p** |
|  | Intercept^*^ | 122.103 ± 1.593 | | 62 | 76.653 | < 0.001 |
|  | Time | -1.394 ± 0.059 | | 62 | -23.661 | < 0.001 |
