# Supplementary material for: Resource Quantity Affects Benthic Microbial Community Structure and Growth Efficiency in a Temperate Intertidal Mudflat
Source: PLoS One. 2012 Jun 18;7(6):e38582. doi: 10.1371/journal.pone.0038582 (PMC3377660; doi:10.1371/journal.pone.0038582)
Supplement: Table S4 — Model output from the DIC concentration data analysis. The optimal model (OM) was a LME model that incorporated core identity as a random effect (L. ratio = 48.237, df1, pcorr<0.001) and allowed the residual spread to increase exponentially over time and to vary by treatment (L. ratio = 179.335, df3, p<0.001): where ai is a random intercept and the index i refers to the core identity (i = 1,…, 9), j to the observations within each core (j = 1,…,6) and k to the treatment (k = 1,…, 3). Random effect (a), variance function (b), correlation coefficients of observations made within each variance grouping (intra-class correlation) and fixed effects (d). *Note the intercept (baseline) is the low diatom-addition treatment. (DOC) [file pone.0038582.s004.doc]

**Table S4. Model output from the DIC concentration data analysis.** The optimal model (OM) was a LME model that incorporated core identity as a random effect (L. ratio = 48.237, df_1_, p_corr_ < 0.001) and allowed the residual spread to increase exponentially over time and to vary by treatment (L. ratio = 179.335, df_3_, p < 0.001):

$$\begin{matrix} Carbon_{ij}=Intercept+Time_{ij}+Treatment_{ij}+ \\ \text{ }Time_{ij}\times Treatment_{ij}+a_{i}+\varepsilon_{ij} \\ a_{i}\sim N(0,\sigma_{Core}^{2}) \\ \varepsilon_{ij}\sim N(0,\sigma_{k}^{2}\times e^{2\delta\times Time_{ij}}) \end{matrix}$$

where *a_i_* is a random intercept and the index *i* refers to the core identity (*i* = 1,..., 9), *j* to the observations within each core (*j* = 1,...,6) and *k* to the treatment (*k* = 1,..., 3). Random effect (a), variance function (b), correlation coefficients of observations made within each variance grouping (intra-class correlation) and fixed effects (d). ^*^Note the intercept (baseline) is the low diatom-addition treatment.

| (a) | **Model term** | **σ** | |  |  |  |
| --- | --- | --- | --- | --- | --- | --- |
|  | Core ID | 0.087 | |  |  |  |
| (b) | **Variance term** | **Variance estimates** | |  |  |  |
|  | δ | 0.105 | |  |  |  |
|  | Low | $0.0016^{2}\times e^{2\times0.105\times Time_{ij}}$ | |  |  |  |
|  | Medium | $(0.0016\times8.082)^{2}\times e^{2\times0.105\times Time_{ij}}$ | |  |  |  |
|  | High | $(0.0016\times107.855)^{2}\times e^{2\times0.105\times Time_{ij}}$ | |  |  |  |
|  |  | **Intra-class correlation** | | |  |  |
| (c) | Time (hrs) | Low | Medium | High |  |  |
|  | 4 | 0.999 | 0.952 | 0.101 |  |  |
|  | 8 | 0.998 | 0.896 | 0.046 |  |  |
|  | 12 | 0.996 | 0.788 | 0.020 |  |  |
|  | 16 | 0.991 | 0.616 | 0.009 |  |  |
|  | 20 | 0.978 | 0.408 | 0.004 |  |  |
|  | 24 | 0.951 | 0.229 | 0.002 |  |  |
| (d) | **Model term** | **Value ± SE** | | **df** | **t** | **p** |
|  | Intercept^*^ | 0.029 ± 0.050 | | 42 | 0.583 | 0.563 |
|  | Medium | -0.038 ± 0.073 | | 6 | -0.523 | 0.620 |
|  | High | -1.003 ± 0.269 | | 6 | -3.735 | 0.010 |
|  | Time | 0.005 ± < 0.001 | | 42 | 18.186 | <0.001 |
|  | Medium×Time | 0.044 ± 0.002 | | 42 | 20.030 | <0.001 |
|  | High×Time | 0.130 ± 0.030 | | 42 | 10.507 | <0.001 |
